# Seven bacterial response-related genes are biomarkers for colon cancer

**DOI:** 10.1186/s12859-023-05204-4

**Published:** 2023-03-20

**Authors:** Zuming Xiong, Wenxin Li, Xiangrong Luo, Yirong Lin, Wei Huang, Sen Zhang

**Affiliations:** Department of Colorectal and Anal Surgery, The First Affiliated Hospital of Guangxi Medical University, No.6 Shuangyong Road, Nanning, 530021 Guangxi Zhuang Autonomous Region People’s Republic of China

**Keywords:** Colon cancer, Bacteria, Prognostic model, Immune

## Abstract

**Background:**

Colon cancer (CC) is a common tumor that causes significant harm to human health. Bacteria play a vital role in cancer biology, particularly the biology of CC. Genes related to bacterial response were seldom used to construct prognosis models. We constructed a bacterial response-related risk model based on three Molecular Signatures Database gene sets to explore new markers for predicting CC prognosis.

**Methods:**

The Cancer Genome Atlas (TCGA) colon adenocarcinoma samples were used as the training set, and Gene Expression Omnibus (GEO) databases were used as the test set. Differentially expressed bacterial response-related genes were identified for prognostic gene selection. Univariate Cox regression analysis, least absolute shrinkage and selection operator-penalized Cox regression analysis, and multivariate Cox regression analysis were performed to construct a prognostic risk model. The individual diagnostic effects of genes in the prognostic model were also evaluated. Moreover, differentially expressed long noncoding RNAs (lncRNAs) were identified. Finally, the expression of these genes was validated using quantitative polymerase chain reaction (qPCR) in cell lines and tissues.

**Results:**

A prognostic signature was constructed based on seven bacterial response genes: *LGALS4, RORC, DDIT3, NSUN5, RBCK1, RGL2, and SERPINE1*. Patients were assigned a risk score based on the prognostic model, and patients in the TCGA cohort with a high risk score had a poorer prognosis than those with a low risk score; a similar finding was observed in the GEO cohort. These seven prognostic model genes were also independent diagnostic factors. Finally, qPCR validated the differential expression of the seven model genes and two coexpressed lncRNAs (C6orf223 and SLC12A9-AS1) in 27 pairs of CC and normal tissues. Differential expression of *LGALS4* and *NSUN5* was also verified in cell lines (FHC, COLO320DM, SW480).

**Conclusions:**

We created a seven-gene bacterial response‐related gene signature that can accurately predict the outcomes of patients with CC. This model can provide valuable insights for personalized treatment.

**Supplementary Information:**

The online version contains supplementary material available at 10.1186/s12859-023-05204-4.

## Background

Colon cancer (CC) is a major human cancer accounting for approximately 10% of all cancer cases [1]. In addition, colorectal cancer (CRC) is a leading cause of cancer and cancer-related deaths worldwide, with a multifactorial etiology that likely includes pro-carcinogenic bacteria [2]. Microbial dysbiosis is a hallmark of CRC and contributes to inflammation, tumor growth, and therapeutic response [3].

The composition of the intestinal microbiota is associated with both tumor development and anticancer immunity. Microbiota-specific T cells have a significant impact on anti-CRC immunity. The introduction of immunogenic intestinal bacteria can promote T follicular helper-associated antitumor immunity in the colon, suggesting therapeutic approaches for the treatment of CRC [4]. Several researchers preliminarily studied the predictive value of bacterial/microbiome-related genes in sepsis, anti-cancer drugs and cancers [5–7]. Metabolism, immune or cell death-related genes have been applied to construct prognostic models for CC or CRC [8–12], but bacterial response-related genes are rarely used as prognostic model to predict prognosis.

The aim of this study was to develop a novel bacterial response prognostic risk score model to provide new insights into the diagnosis, evaluation, and treatment of CC.

## Results

### Bacterial response-related differentially expressed genes (DEGs) in CC samples

**Fig. 1:**
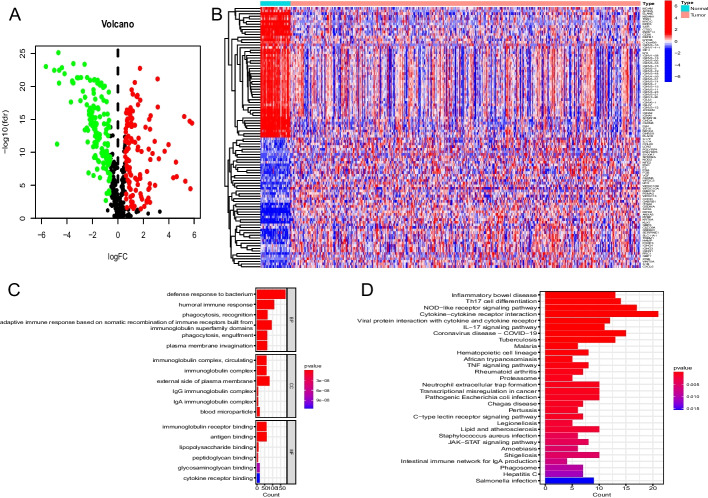
**A** Volcano map of bacterial response-related genes (red: upregulated genes, green: downregulated genes). **B** Differential expression heatmap between CC and normal colon tissues in the TCGA database. **C** GO analysis of 276 DEGs. **D** KEGG pathways analysis of 276 DEGs

We examined DEGs between normal colon and CC tissue using The Cancer Genome Atlas (TCGA) database. A total of 276 statistically significant differentially regulated genes were identified (Fig. 1A, Additional file 2: Table S1). Of these, 124 genes were increased and 152 were decreased in CC samples. Figure 1B shows the top 50 upregulated and downregulated bacterial response-related DEGs. 276 DEGs mianly enriched in defense response to bacterium and other Gene Ontology (GO) terms (Fig. 1C). They also enriched in many Kyoto Encyclopedia of Genes and Genomes (KEGG) pathways,including Coronavirus disease—COVID-19, Tuberculosis, Pathogenic Escherichia coli infection, Staphylococcus aureus infection, Salmonella infection and so on(Fig. 1D).

### Prognostic risk score model construction in the TCGA cohort

**Fig. 2:**
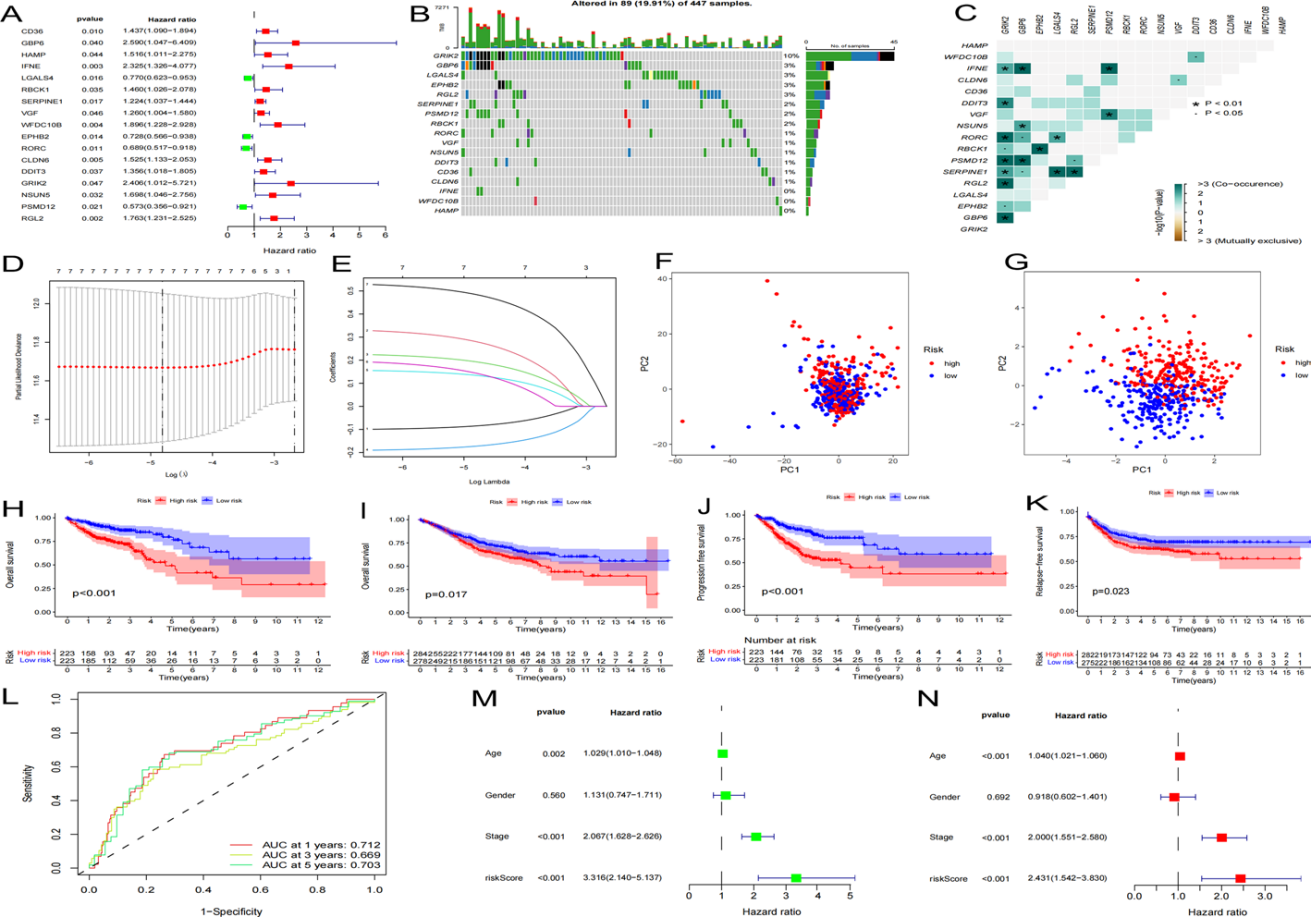
Prognostic risk score model construction. **A** Forest plot of 17 bacterial response-related DEGs associated with prognosis. **B** The mutation frequency of the 17 bacterial response-related DEGs in 447 CC patients from TCGA. **C** Mutational co-occurrence (green) and exclusion (purple) analyses of the 17 bacterial response-related DEGs. **D** LASSO coefficients of the seven bacterial response-related DEGs. **E** Construction of the prognostic risk score model. **F** PCA of all bacterial response-related genes in the training set. **G** PCA of the bacterial response risk score to separate CC samples from normal samples in the training set; red indicates patients with a high risk score, and blue indicates patients with a low risk score. **H** and **I** OS of training and test sets in the two risk score groups. **J** Progression-free survival of the training set in the two risk score groups. **K** Relapse-free survival of the test set in the two risk score groups. **L** ROC curves of the predictive efficiency in the training set. **M** and **N** Forest plots of univariate and multivariate Cox regression analyses in the training set

TCGA cohort samples were used as the training set to construct a prognostic model. Univariate Cox regression analysis was applied to the 276 bacterial response-related DEGs. Seventeen genes were related to prognosis (all *p* < 0.05) (Fig. 2A). The somatic mutation profiles of the 17 genes were collected. Mutations in these bacterial response-related genes were detected in 89 of 447 CC samples (frequency: 19.91%, Fig. 2B). *GRIK2* had the highest mutation frequency; no mutations were identified in *IFNE, WFDC10B,* or *HAMP* in any of the CC samples. Further analyses demonstrated that most of these 17 genes had mutational co-occurrence relationships (Fig. 2C).

Expression requirements and least absolute shrinkage and selection operator (LASSO) Cox regression analyses were utilized together to decrease the number of genes incorporated into the model. Finally, seven genes (*LGALS4, RORC, DDIT3, NSUN5, RBCK1, RGL2, and SERPINE1*) were included in the prognostic risk score model (Figs. 2D and E). The following formula was used to calculate the risk score of each sample: risk score = (− 0.0849135799481108) × LGALS4 + (− 0.172643229881538) × RORC + (0.134531384115045) × DDIT3 + (0.144199163862461) × NSUN5 + (0.280831773407297) × RBCK1 + (0.484753562928209) × RGL2 + (0.193242156693903) × SERPINE1.

The median risk score in the training set was set as the cutoff value. Based on each patient’s calculated risk score, 446 patients each were allocated to the low and high risk score groups. Principal-component analysis (PCA) results showed that the risk score had good discrimination in CC (Figs. 2F and G). Patients in the high risk score group had a poorer overall survival (OS) and progression-free survival (PFS) than those in the low risk score group (Figs. 2H and J). The 1-, 3-, and 5-year time-dependent receiver operating characteristic (ROC) curves confirmed that the prognostic risk score model had good predictive performance (area under the ROC curves [AUCs] of 0.712, 0.669, and 0.703, respectively; Fig. 2L). Figures 2M and N indicate that the risk score can serve as an independent risk indicator.

We next validated this prognostic model in the test set (GSE39582). These patients were also divided into low and high risk score groups using the cutoff value from the training set, OS and relapse-free survival (RFS) prognosis were assessed (Fig. 2I and K). Patients in the high risk score group had worse prognosis than those in the low risk score group, demonstrating that the prognostic risk score model had good OS PFS and RFS predictive ability in CC across multiple datasets. The 1-, 3-, and 5-year time-dependent ROC curves, univariate and multivariate Cox regression analyses were also assesed in the test set (Additional file 1: Fig. S1A–C).

### Construction of a nomogram for predicting survival

**Fig. 3:**
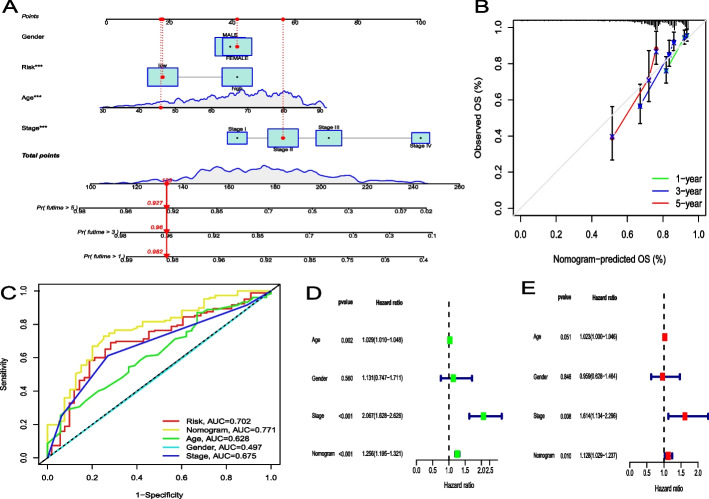
The predictive value of the nomogram. **A** Nomogram predicting the OS of CC patients in TCGA. **B** Calibration plot of the nomogram. The nomogram-predicted survival is displayed on the x-axis, and the actual survival is displayed on the y-axis. **C** ROC curves for the risk score and clinicopathological characteristics. **D**, **E** Univariate and multivariate Cox regression analyses of factors associated with OS

Figure 3A shows the OS predictive nomogram combining age, sex, pathological stage, and the prognostic risk score model. The 1-, 3-, and 5-year calibration curves confirmed the ability of the nomogram to predict OS in CC patients (Fig. 3B). The AUC of the nomogram was 0.771, indicating that it had better prognostic performance than age (AUC = 0.628), pathological stage (AUC = 0.675), or the prognostic risk score model (AUC = 0.702; Fig. 3C). In addition, the nomogram model was an independent prognostic factor for OS (Figs. 3D and E).

### Association between the risk score and clinical characteristics

**Fig. 4:**
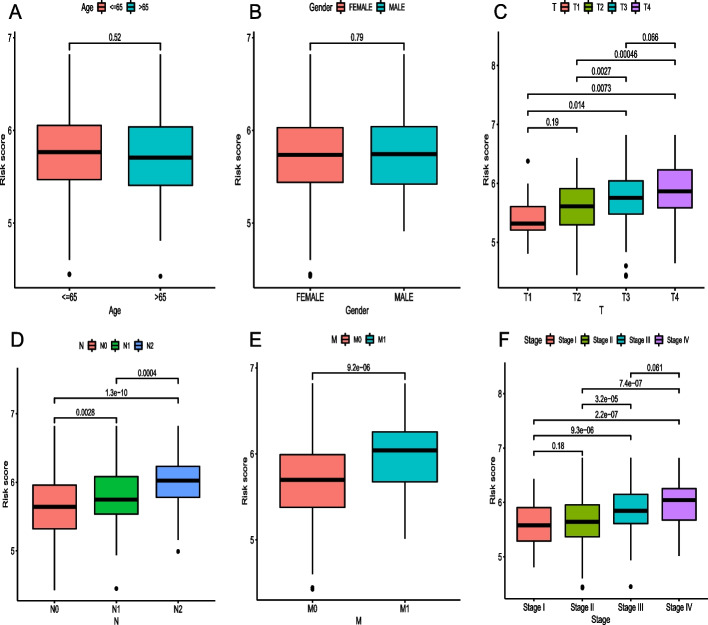
**A** − **F** The association of the risk score with clinicopathological features, including age, sex, and T, N, M, and TNM stages

The distribution of risk scores based on age, sex, pathological stage, and American Joint Committee on Cancer (AJCC) Tumor Node Metastasis (TNM) classification of malignant tumors stage [8] of the corresponding samples was analyzed. The risk score had no significant associations with age or sex (Fig. 4A, B). However, risk scores were consistently correlated with four clinical characteristics: T (tumor invasion), N (lymphoid metastasis), and M (distal metastasis) stages, as well as advanced pathological stage (all *p* < 0.05, Fig. 4C–F).

### Response to chemotherapy

**Fig. 5:**
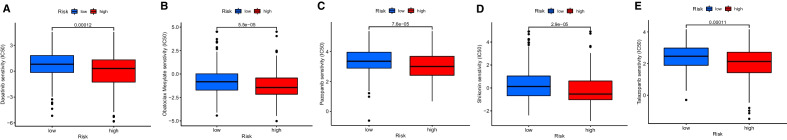
**A**–**E** Differential chemotherapeutic response based on the IC50s in the high and low risk score groups. IC50s of five chemotherapeutic agents (dasatinib, obatoclax mesylate, pazopanib, shikonin, and talazoparib)

The pRRophetic R package was used to evaluate differences in chemosensitivity between the two risk groups in the training set. Figure 5A–E shows the calculated half-maximal inhibitory concentrations (IC50s) of several traditional anticancer drugs, including dasatinib, obatoclax mesylate, pazopanib, shikonin, and talazoparib, in the two risk score groups. These five drugs had lower IC50s in the high risk score group, which is suggestive of better efficacy.

### Gene set variation analysis (GSVA) and TP53 mutation

**Fig. 6:**
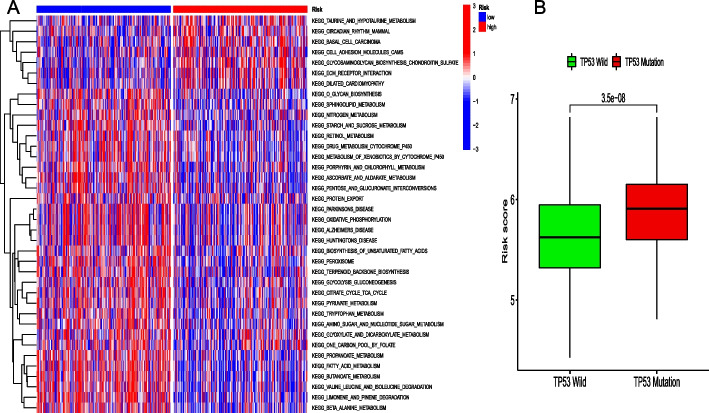
**A** The heatmap of GSVA enrichment in the low and high risk score groups. **B** Differences in risk scores in *TP53* wild-type and mutant samples

Differences in biological behaviors between the high and low risk score groups were examined using GSVA enrichment. Basal cell carcinoma was enriched in the high risk score group. However, most metabolic pathways, including butanoate metabolism, fatty acid metabolism, sphingolipid metabolism, and starch and sucrose metabolism, were enriched in the low risk score group (Fig. 6A). Furthermore, CC patients with *TP53* mutations had higher risk scores (Fig. 6B).

### Immune-related features in the low and high risk score groups

**Fig. 7:**
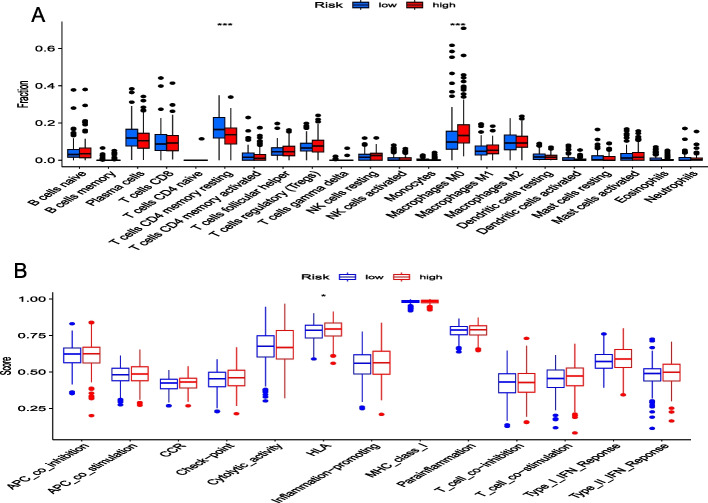
**A** Differences in infiltration of immune cells between the low and high risk score groups. **B** Differences in known immunity-related functions between the low and high risk score groups (*p < 0.05)

The high risk score group was rich in M0 macrophages. However, the low risk score group was rich in CD4 memory resting T cells (Fig. 7A). Moreover, HLA was abundant in the high risk score group, suggesting that patients with a high risk score and immune suppression may benefit from immunotherapy (Fig. 7B).

### GO and KEGG pathway analyses and protein–protein interaction (PPI) networks of DEGs in the low and high risk score groups

**Fig. 8:**
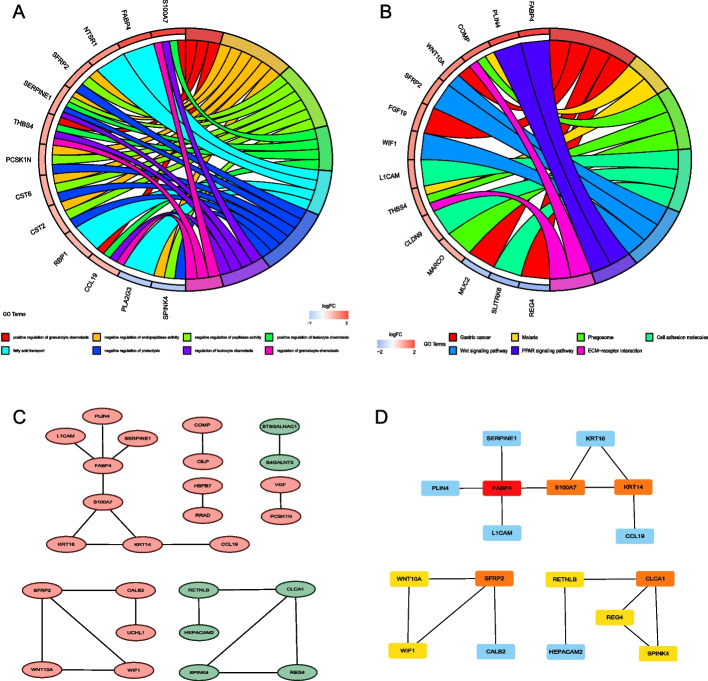
**A** and **B** GO and KEGG enrichment analyses of 60 DEGs in the low and high risk score groups. **C** PPI network processed by Cytoscape. Red: DEGs with high expression in the high risk score group; green: DEGs with high expression in the low risk score group. **D** Top 10 hub genes in cytoHubba analysis (red and yellow)

GO and KEGG pathway analyses of 60 DEGs were performed using the “GOplot” R package to better explore the function of these DEGs (Additional file 3: Table S2). The results of the GO analysis indicated that DEGs participated in the positive regulation of granulocyte chemotaxis, negative regulation of endopeptidase activity, negative regulation of peptidase activity, positive regulation of leukocyte chemotaxis, fatty acid transport, negative regulation of proteolysis, regulation of leukocyte chemotaxis, and regulation of granulocyte chemotaxis (Fig. 8A).

The KEGG pathway analysis indicated that DEGs were enriched in gastric cancer, malaria, phagosome, cell adhesion molecules, Wnt signaling pathway, PPAR signaling pathway, and ECM-receptor interaction (Fig. 8B).

The DEGs in the high and low risk score groups were analyzed using the STRING online database. The PPI network shown in Additional file 1: figure S2 was plotted using DEGs. These DEGs in Fig. 8 C and D may have protein–protein interactions. Figure 8C shows DEG interactions, where red indicates genes increased in the high risk score group and green indicates genes increased in the low risk score group. Figure 8D shows the 10 hub genes selected in the network, including *CLCA1, FABP4, KRT14, REG4, RETNLB, S100A7, SFRP2, SPINK4, WIF1,* and *WNT10A*. *REG4* and *S100A7* were upregulated in CC samples compared to normal colon samples, whereas *CLCA1, FABP4, KRT14, RETNLB, SFRP2,* and *WNT10A* were downregulated in CC samples (Additional file 1: Fig. S3).

K- M analysis indicated that *REG4, S100A7, CLCA1, FABP4, RETNLB, SFRP2,* and *WNT10A* expressions were significantly related to CC patient prognosis (Additional file 1: Fig. S4).

### Expression, diagnostic value, and prognostic value of the seven prognostic model genes

**Fig. 9:**
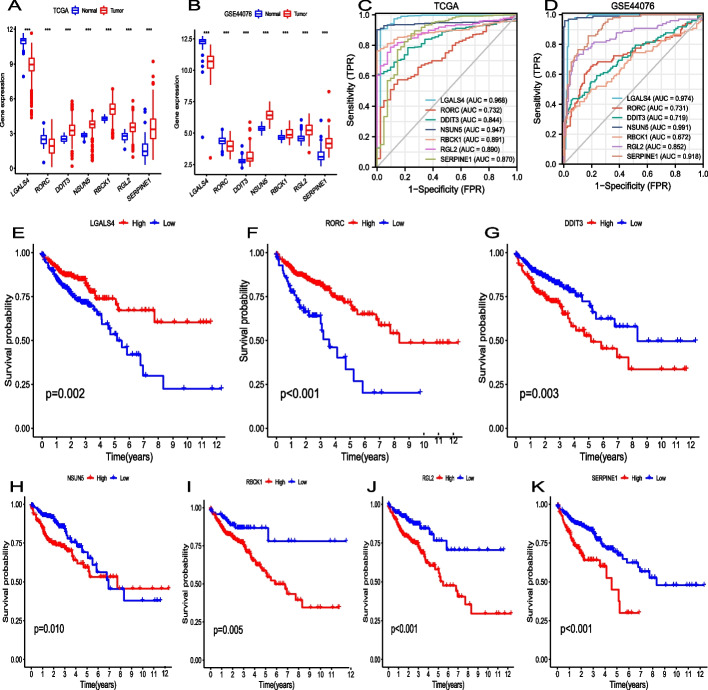
**A** Expression of the seven prognostic model genes in normal and cancer tissue samples in the TCGA database. **B** Expression of the seven prognostic model genes in GSE44076 (98 CC tissues and 148 normal colon tissues). **C** and **D** Diagnostic ROC curves for the expression of each prognostic gene in the TCGA and GSE44076 datasets between CC tissues and normal colon tissues. **E**–**K** Kaplan–Meier curves of OS for the seven prognostic model genes in TCGA. (***p < 0.001)

We further characterized the seven prognostic model genes in the TCGA and GEO datasets. *DDIT3, NSUN5, RBCK1, RGL2,* and *SERPINE1* were increased in CC samples compared to normal samples in the TCGA cohort, whereas *LGALS4* and *RORC* were decreased in CC samples (Fig. 9A). The same trend was observed in the GSE44076 dataset (Fig. 9B). In the TCGA cohort and GSE44076 dataset, the expression of the seven prognostic model genes could distinguish cancer tissue from normal tissue (Figs. 9C, D). *LGALS4* and *NSUN5* had the best accuracy in differentiating CC and normal tissues (AUCs: TCGA, *LGALS4*, 0.968 and *NSUN5,* 0.947; GSE44076, *LGALS4,* 0.974 and *NSUN5,* 0.991). Moreover, each of the seven prognostic model genes could be individually used to stratify OS (Fig. 9E–K).

### Identification of coexpressed prognostic long noncoding RNAs (lncRNAs)

**Fig. 10:**
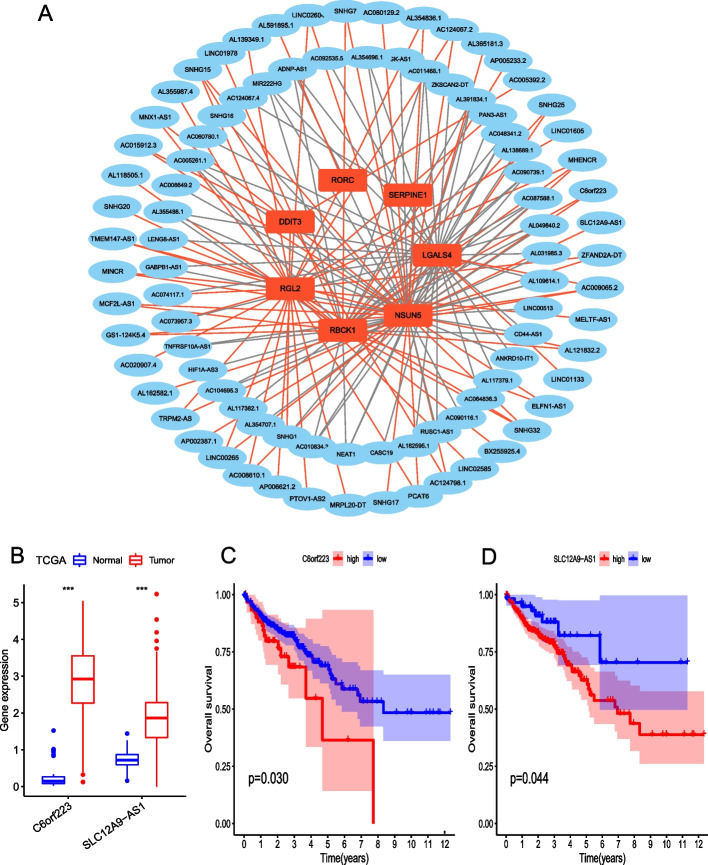
**A** Ninety lncRNAs were coexpressed with the seven prognostic model genes. Gray lines indicate negative correlation; red lines indicate positive correlation. **B** Expression of the coexpressed lncRNAs C6orf223 and SLC12A9-AS1 in TCGA. **C** and **D** Kaplan–Meier curves of OS based on C6orf223 and SLC12A9-AS1 expression in TCGA. (***p < 0.001)

A total of 1832 significantly differentially expressed lncRNAs were identified in the TCGA cohort; 1594 lncRNAs were upregulated in CC samples and 238 were downregulated in CC samples (Additional file 4: Table S3). A total of 999 lncRNAs were significantly associated with OS, of which 90 were coexpressed with the seven prognostic model genes (Fig. 10A), including C6orf223 and SLC12A9-AS1 (Figs. 10B–D).

### qPCR validation in cell lines and colon tissues

**Fig. 11:**
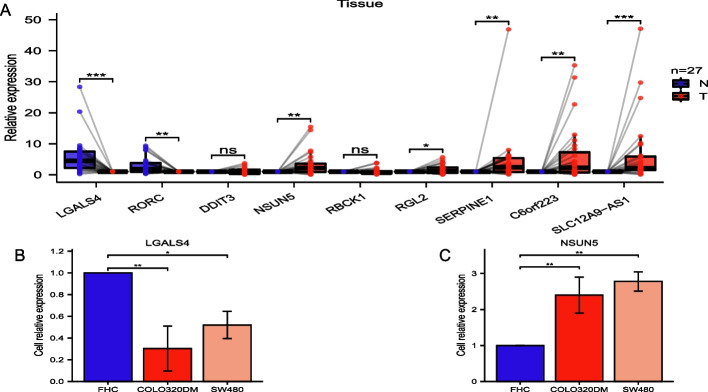
The bioinformatics results were validated using quantitative PCR in CC tissues and cell lines. **A** Relative expression of the seven prognostic model genes and their two coexpressed lncRNAs in CC and normal colon tissues. **B, C** Relative expression of *LGALS4* and *NSUN5* in three cell lines (FHC, COLO320DM, and SW480). (*p < 0.05, **p < 0.001, ***p < 0.001)

The expression of the seven prognostic genes and two prognostic lncRNAs was verified in 27 CC and matched normal tissues. *NSUN5, RGL2, SERPINE1,* C6orf223, and SLC12A9-AS1 had higher expression in CC tissues than in normal tissues, whereas *LGALS4* and *RORC* had lower expression in CC tissues (all *p* < 0.05; Fig. 11A). This was consistent with the results of the bioinformatics analysis. *NSUN5* expression was higher in COLO320DM and SW480 than in FHC (Fig. 11C), and *LGALS4* expression was lower in COLO320DM and SW480 (Fig. 11B).

## Discussion

The interactions and relationships between CC and bacteria are complex. Chronic and low-grade inflammation associated with persistent bacterial infections have been linked to the development of colon tumors [13]. Each tumor type may have a specific microbiome. Intratumoral bacteria are mostly intracellular and present in both cancer and immune cells. Manipulation of the tumor microbiome may also affect tumor immunity and response to immune therapy. Therefore, better understanding bacterial response effects may pave the way for novel treatment options for cancer patients [14]. Previous studies found that antimicrobial genes had strong correlation with sepsis and may predict sepsis [5]; bacterial infection related genes may predict safety and efficacy of Immunotherapy [6]. Bacterial infection is one kind of common infection inducing immune. Immune—related genes have been applied to construct prognostic models for CC [12]. Our seven-gene bacterial response‐related prognostic model can accurately predict the OS of CC patients.

We used data from the TCGA database to create a prognostic risk score model to predict the OS of CC patients. Our risk model included five genes expressed at high levels in cancer tissues and two genes expressed at low levels in cancer tissues. Patients with CC in the high risk score group had poorer OS than those in the low risk score group. The same result was obtained using a dataset from the GEO database, demonstrating that the prognostic risk score model can predict patient survival. A high risk score was also associated with more advanced T, N, M, and TNM stages. Further, these seven genes could also independently predict the prognosis and diagnosis of CC.

By comparing immune status and response to therapy between the low and high risk score groups, we also showed that the prognostic risk score model can also distinguish immune and therapeutic differences in CC. Patients with a high risk score were also more likely to have mutated *TP53*.

Owing to the significant differences between the low and high risk score groups, DEGs in the two groups were further studied. Seven hub genes, *REG4, S100A7, CLCA1, FABP4, RETNLB, SFRP2,* and *WNT10A*, were all significantly associated with CC patient prognosis and differentially expressed in normal and tumor tissues.

Previous studies had also demonstrated that these prognostic model genes are closely related to diseases. *LGALS4* is associated with multiple cancer types and is a possible prognostic and diagnostic marker of colon adenocarcinoma (COAD) [15]. This is consistent with our findings. *LGALS4* is expressed at significantly higher levels in adjacent normal tissues than in CC tissues [16], and it has been suggested to function as a tumor suppressor in CRC [17]. *RORC* is a critical transcription factor for the generation of pro-inflammatory cytokines, which are closely related to the pathogenesis of autoimmune diseases [18]. *RORC* is also closely related to inflammatory reaction and colitis [19, 20]. *DDIT3* is a key stress response regulator activated in diverse settings, including DNA damage, ER stress, hypoxia, and nutrient deprivation [21, 22]. *DDIT3* also plays an important role in the integrated stress response [23]. The summary actions of *DDIT3* increase the survival of cancer cells when exogenous glutamine is limited [21]. *NSUN5* is a conserved RNA methyltransferase that belongs to the Nop2/SUN domain family. The loss of *NSUN5* decreases growth, cell size, proliferation, and bulk protein translation [24]. *NSUN5* is overexpressed in CRC, and *NSUN5* increases CRC proliferation and progression mostly through cell cycle regulation. Knockdown of *NSUN5* inhibits tumor growth in vivo and in vitro [25]. High expression of RBCK1 is associated with poorer overall outcomes in patients with CRC. Moreover, *RBCK1* suppression markedly reduces stemness in CRC [26]. *RGL2* is significantly upregulated in primary tumors compared to normal tissues and serves as a poor prognostic marker in patients with CRC. Cell-based and animal experiments have further demonstrated that *RGL2* acts as a driver to promote the metastatic progression of CRC, most likely by preventing the degradation of β-catenin and *KRAS *[27]. *SERPINE1* is associated with cell migration and cell death. Increased *SERPINE1* expression induces the expression of *MMP1* and increases cell motility. The administration of agents that inhibit *SERPINE1* should also be considered to reduce the risk of cancer cell metastasis [28].

In a previous study, C6orf223 (also known as LINC03040) was confirmed to be continuously upregulated in CC tissues compared to normal colon tissues [29]. Our study also confirmed that C6orf223 was significantly upregulated in CC tissues and predicted poor prognosis for patients with CC. In addition, we demonstrated that SLC12A9-AS1 was highly expressed in CC tissues by qPCR. However, the potential mechanism by which C6orf223 and SLC12A9-AS1 affect the prognosis of CC patients remains unclear.

Our study has some limitations. First, our results were mainly based on bioinformatics analysis, and further in vivo and in vitro experiments are needed. Second, some factors that may affect CC prognosis were not examined, including weight, diet, and family history [30]. Finally, there was no significant difference in *DDIT3* or *RBCK1* expression between the two risk score groups, which may be due to a bias in the sample distribution.

## Conclusions

Our seven-gene signature based on bacterial response-related genes demonstrated favorable predictive ability in both the training and test sets. The seven genes were all independent prognostic and diagnostic factors. Therefore, we recommend this signature to evaluate the prognostic risk of patients with CC.

## Methods

### Datasets

COAD patient genomic data were downloaded from the TCGA database (https://portal.gdc.cancer.gov/). Gene expression data from 41 normal and 473 CC cases were selected for the analyses. The corresponding clinical data and somatic mutation data were obtained from the TCGA database. The external dataset for the prognostic test set of 562 CC patients was derived from the GEO database (https://www.ncbi.nlm.nih.gov/geo/) (GSE39582). Data from 98 CC tissues and 148 normal colon tissues for the diagnostic test set was obtained from GSE44076. The expression data was standardized to mRNA and lncRNA transcript fragments per kilobase million using Perl software.

We searched the keywords “bacteria & bacterium” in the Molecular Signatures Database (MSigDB) (https://www.gsea-msigdb.org/gsea/msigdb/human/search.jsp) [31–34]. Three GSEA gene sets (GOBP_DEFENSE_RESPONSE_TO_BACT- ERIUM; GOBP_T_HELPER_17_TYPE_IMMUNE_RESPONSE; GSE20151_CTRL_VS_FUSOBACT_NUCLEATUM_NEUTROPHIL_DN) and 611 bacterial response- related genes were identified (Additional file 5: Table S4).

### Prognostic risk score model construction and verification

Bacterial response-related genes that were differentially expressed between normal and cancer tissue samples were identified using the “limma” R package (|logFC|> 0.585 and false discovery rate [FDR] < 0.05).

The TCGA cohort samples were used as the training set, and GSE39582 samples were used as the test set. The differentially expressed bacterial response-related genes were combined with the corresponding prognosis results. These DEGs wrer conducted GO and KEGG enrichment analyses (*p* < 0.05) [35–38]. Intersecting differentially expressed bacterial response-related genes in the training and test sets were identified. Prognostic genes were identified from the intersecting genes using univariate Cox regression analysis of the training set, and genes with *p* < 0.05 were selected.

The mutations and correlations of the prognostic genes of the training set CC samples were analyzed using the Maftools package in R.

Seven genes were chosen for LASSO Cox regression, which requires that the average gene expression of cancer and normal is greater than 1, and the average expression of high-risk genes is higher in the cancer group, whereas the average expression of low-risk genes is higher in the normal group. Then, based on these seven genes, prognostic risk score model was constructed to predict OS. The following equation was performed to calculate the risk score of each sample:

$${\text{Risk}}\;{\text{score}} = \sum\nolimits_{1}^{i} {({\text{Coefi }} \times {\text{ExpGenei}})}$$

Finally, the median risk score was used to divide patients into the low and high risk score groups.

The OS and PFS differences in low- and high-risk score groups were analyzed. The predictive accuracy of the prognostic risk score mode was estimated using the “survivalROC” package in R. Finally, the same procedures were performed in the test set.

### PCA comparison before and after construction of the prognostic risk score model

The PCA difference in the two risk score groups was identified using the “limma” R package. PCA was performed and displayed using the ggplot2 package before and after construction of the prognostic model.

### Nomogram construction for predicting OS

The “rms” R package was used to construct the OS predictive nomogram in CC patients, including age, sex, pathological stage, and the prognostic risk score model. Time-dependent calibration curves were drawn and AUCs were calculated to predict the accuracy of the nomogram. Univariate and multivariate Cox regression analyses were analyzed.

### Relationship between risk scores and clinical characteristics

The CMScaller package in R was used to divide all the samples into consensus molecular subtypes according to their features in the training set. The Limma package in R was further utilized to screen the relationship between risk scores and sex, age, pathological stage, and AJCC TNM stage (*p* < 0.05).

### GSVA

The “GSVA” R package was used to perform GSVA to compare differences in biological processes between gene profiles in the low and high risk score groups (FDR < 0.05). GSVA is a non-parametric and unsupervised method to distinguish pathway variations or biological processes through an expression matrix sample [39]. The reference gene set came from the “c2.cp.kegg.v7.4. symbols” gene set in MSigDB (https://www.gsea-msigdb.org/gsea/msigdb).

### Characteristic differences between the low and high risk score groups

The “pRRophetic” package in R was used to evaluate the chemosensitivity dasatinib, obatoclax mesylate, pazopanib, shikonin, and talazoparib in the low and high risk groups in the training set (*p* < 0.001). The IC50 indicates the ability of a substance to inhibit certain biological or biochemical functions [8].

The immune-related infiltration of each training set sample was performed through ssGSEA. A previously verified gene set was utilized to evaluate the immune-related characteristics in the tumor microenvironment, including different immune-related activities and human immune cell subtypes, such as B cells, CD4 + T cells, and macrophages [8, 40, 41]. The ssGSEA algorithm was used to calculate enrichment scores and analyze the relationship between the risk score and immune-related characteristics.

### GO and KEGG analyses and PPI network construction

The “limma” R package was used to compare the RNA-seq data profiles of the low and high risk score groups. DEGs in the low and high risk score groups were identified (adjusted *p* < 0.05), and the “clusterProfiler” R package was used to conduct GO and KEGG enrichment analyses of DEGs (*p* < 0.05).

The STRING online database (version 11.5; https://string-db.org/) was used to analyze the DEGs to create a PPI network with median confidence (interaction score > 0.40). Cytoscape software (version 3.9.1) was used to plot the PPI network. Hub genes from all DEGs were searched using cytoHubba (Cytoscape plug-in, version 0.1) and topological algorithms.

### Expression of the seven prognostic model genes and lncRNAs

The “limma” R package was used to analyze differential expression of the seven prognostic model genes in the TCGA and GSE44076 datasets. LncRNAs were also analyzed using R in normal colon and CC tissue samples. LncRNAs with |logFC|> 1 and FDR < 0.05 were considered statistically significant.

### Identification of lncRNAs associated with the seven prognostic model genes

Kaplan–Meier analysis was used to batch filter the prognostic lncRNAs. R was used to further evaluate the association between the expression of lncRNAs and the seven prognostic model mRNAs in CC samples. The association was determined using Pearson’s correlation coefficient analysis (lncRNA mean > 1, *p* < 0.001, |correlation coefficient|> 0.2). Finally, the co-expression network data were analyzed and plotted using Cytoscape software.

### qPCR

*LGALS4, RORC, DDIT3, NSUN5, RBCK1, RGL2,* and *SERPINE1* and their coexpression with lncNRAs C6orf223 and SLC12A9-AS1 were verified in Twenty-seven pairs of matched adjacent normal and CC tissue samples. Informed consent was obtained from all the participants. Cell lines FHC, COLO320DM, SW480 were used for verifing *LGALS4* and *NSUN5* expressions. All aspects of this study were approved by the Ethics Committee of the First Affiliated Hospital of Guangxi Medical University (Aproval Number: 2022-E415-01, detailed experimental methods and qPCR primers are in Additional file 6).

### Statistical analysis

Differences between the two groups were compared using the Wilcoxon test. One-way analysis of variance, Welch one-way ANOVA  or K-W test was used to compare three or more groups. All statistical analyses were performed using R, and *p* < 0.05 was considered statistically significant.

## Supplementary Information

**Additional file 1: Figure S1.** **A** ROC curves of the predictive efficiency in the test set. **B** and **C** Forest plots of univariate and multivariate Cox regression analyses in the test set. **Fig. S2.** PPI network of DEGs in the high and low risk score groups. **Fig. S3.** Expression of REG4， S100A7， CLCA1, FABP4, KRT14, RETNLB, SFRP2, and WNT10A in TCGA CC and normal colon samples. **Fig. S4.** **A**–**G** OS of REG4, S100A7, CLCA1, FABP4, RETNLB, SFRP2, and WNT10A in TCGA.

**Additional file 2: Table S1.** 276 differentially expressed genes between normal and colon cancer samples in TCGA.

**Additional file 3: Table S2.** Differentially expressed genes in the low and high risk score groups.

**Additional file 4: Table S3.** Differentially expressed lncRNAs of TCGA CC samples.

**Additional file 5: Table S4.** Total 611 bacterial response-related genes from 3 GSEA gene sets.

**Additional file 6.** qPCR methods and the primers (Sangon) used for 9 genes qPCR.

**Additional file 7.** Data or codes for figure 4–7.

## Data Availability

All data are available in TCGA (https://portal.gdc.cancer.gov/), GEO (https://www.ncbi.nlm.nih.gov/geo/. GEO accession: GSE39582, GSE44076), GSEA and other corresponding databases. The data or codes of Figs. 4–7 are included in Additional file 7.The analysis methods used in the current study can be obtained from the corresponding authors according to reasonable requirements.

## Abbreviations

AJCC: American Joint Committee on Cancer
AUC: Area under the ROC curve
CC: Colon cancer
*CLCA1*: Chloride channel accessory 1
COAD: Colon adenocarcinoma
CRC: Colorectal cancer
*DDIT3*: DNA damage induced transcript 3
DEG: Differentially expressed gene
*FABP4*: Fatty acid binding protein 4
FDR: False discovery rate
GEO: Gene Expression Omnibus
GO: Gene Ontology
GSEA: Gene set enrichment analysis
GSVA: Gene set variation analysis
IC50: Half-maximal inhibitory concentrations
KEGG: Kyoto Encyclopedia of Genes and Genomes
*KRT14*: Keratin 14
LASSO: Least absolute shrinkage and selection operator
*LGALS4*: Galectin 4
lncRNA: Long noncoding RNA
MSigDB: Molecular Signatures Database
*NSUN5*: NOP2/Sun RNA methyltransferase 5
OS: Overall survival
PFS: Progression-free survival
PPI: Protein–protein interaction
*RBCK1*: RANBP2-type and C3HC4-type zinc finger containing 1
*REG4*: Regenerating family member 4
*RETNLB*: Resistin-like beta
*RGL2*: Ral guanine nucleotide dissociation stimulator like 2
ROC: Receiver operating characteristic
*RORC*: Retinoic acid receptor-related orphan receptor gamma
*S100A7*: S100 calcium binding protein A7
*SERPINE1*: Serpin family E member 1
*SFRP2*: Secreted frizzled related protein 2
TCGA: The Cancer Genome Atlas
TNM: Tumor node metastasis
*WNT10A*: Wnt family member 10A
