# Supplementary material for: Seven bacterial response-related genes are biomarkers for colon cancer
Source: BMC Bioinformatics. 2023 Mar 20;24:103. doi: 10.1186/s12859-023-05204-4 (PMC10026208; doi:10.1186/s12859-023-05204-4)

Figure S1 (A)ROC curves of the predictive efficiency in the test set. (B and C) Forest plots of univariate and multivariate Cox regression analyses in the test set.


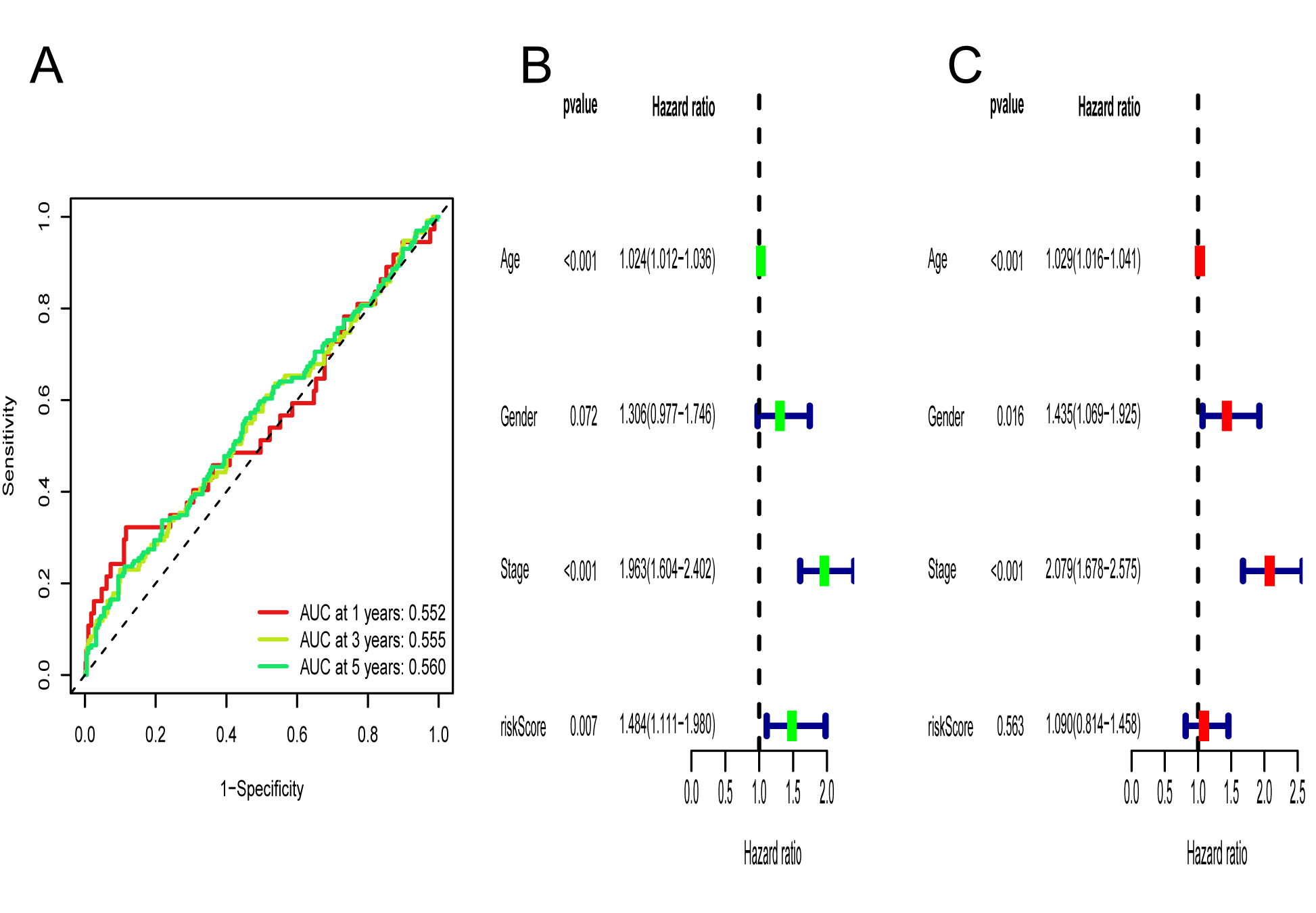


Figure S2 PPI network of DEGs in the high and low risk score groups


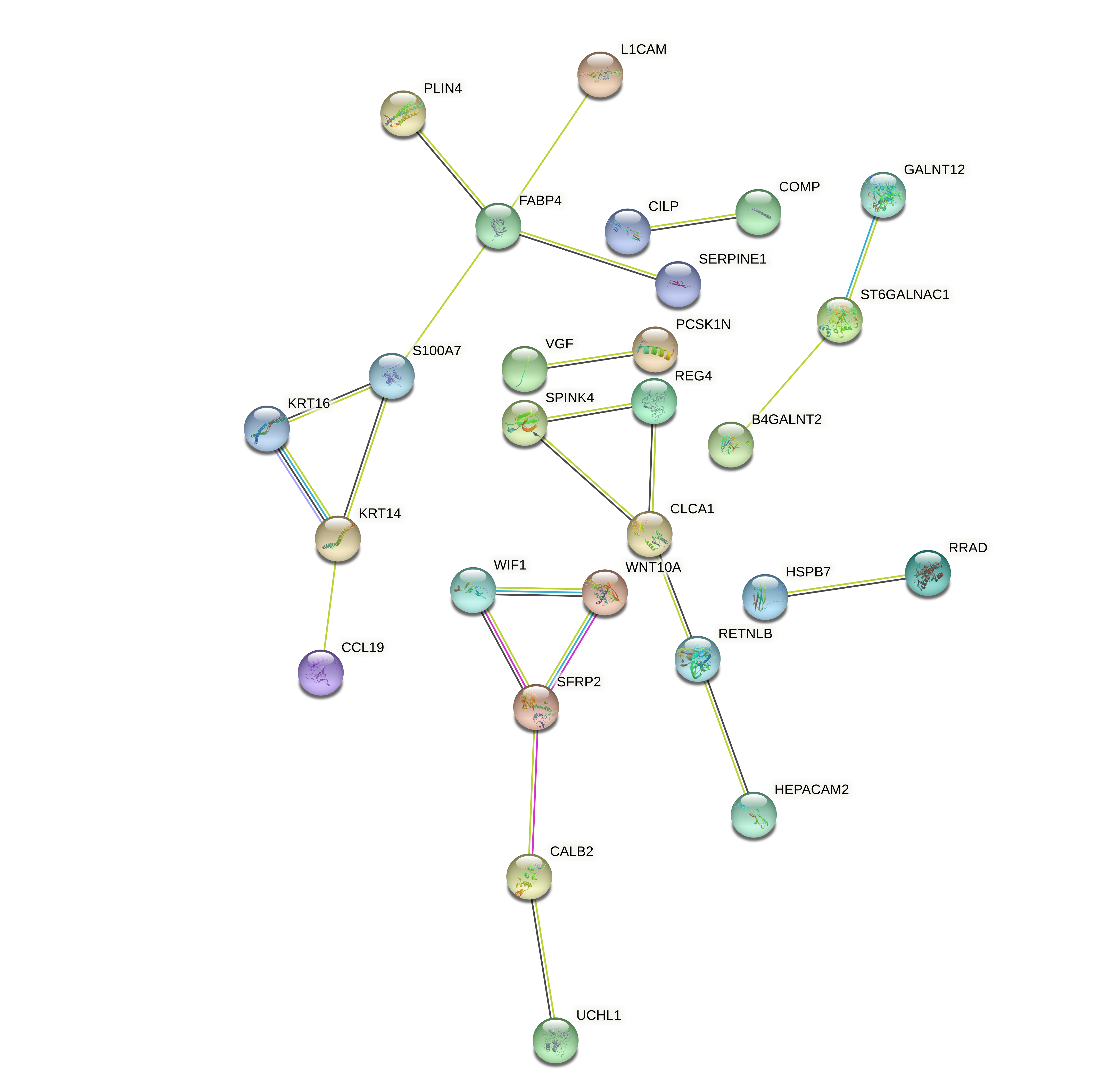


Figure S3 Expression of REG4， S100A7， CLCA1, FABP4, KRT14, RETNLB, SFRP2, and WNT10A in TCGA CC and normal colon samples


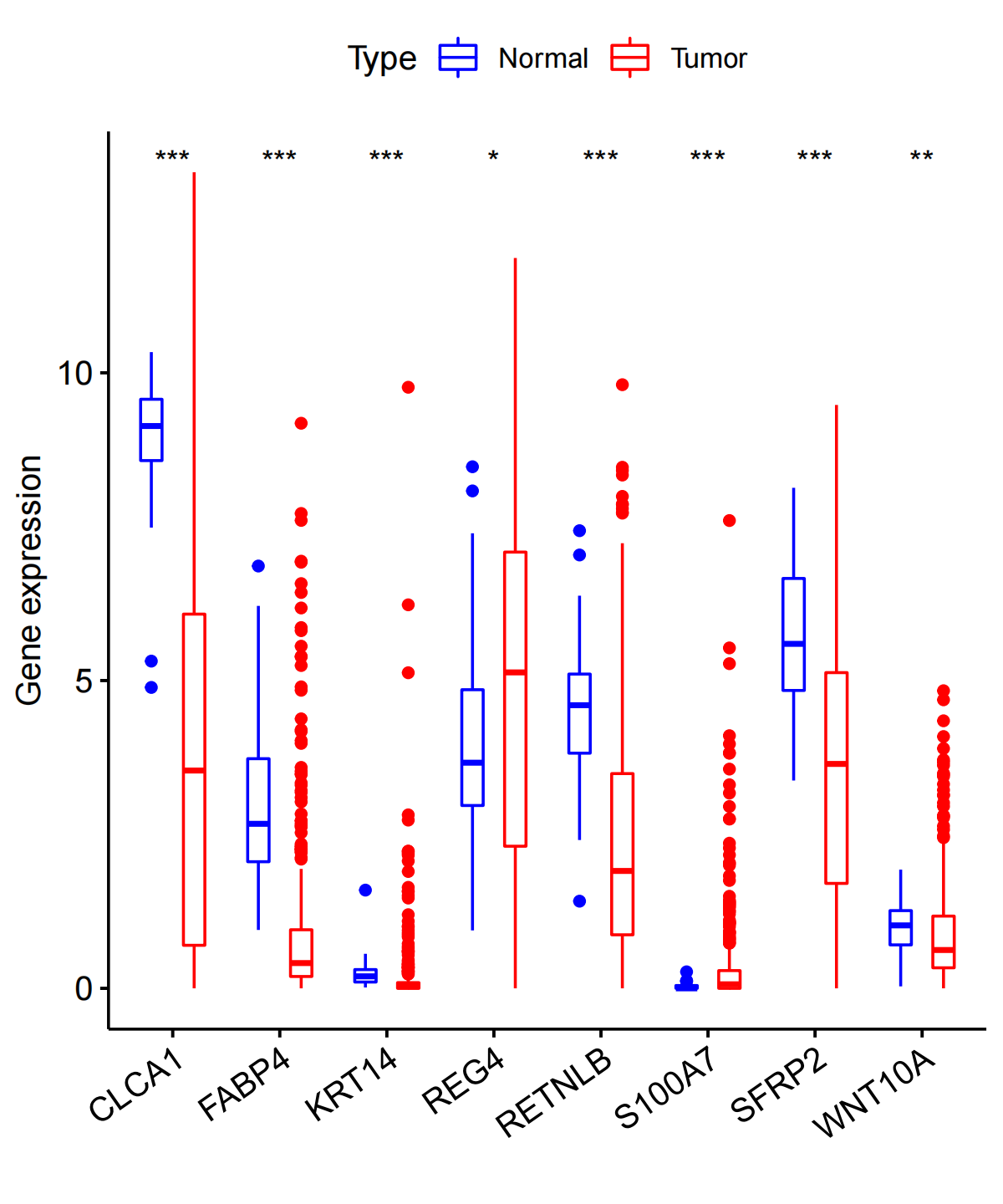


Figure S4 (A-G) OS of REG4, S100A7, CLCA1, FABP4, RETNLB, SFRP2, and WNT10A in TCGA


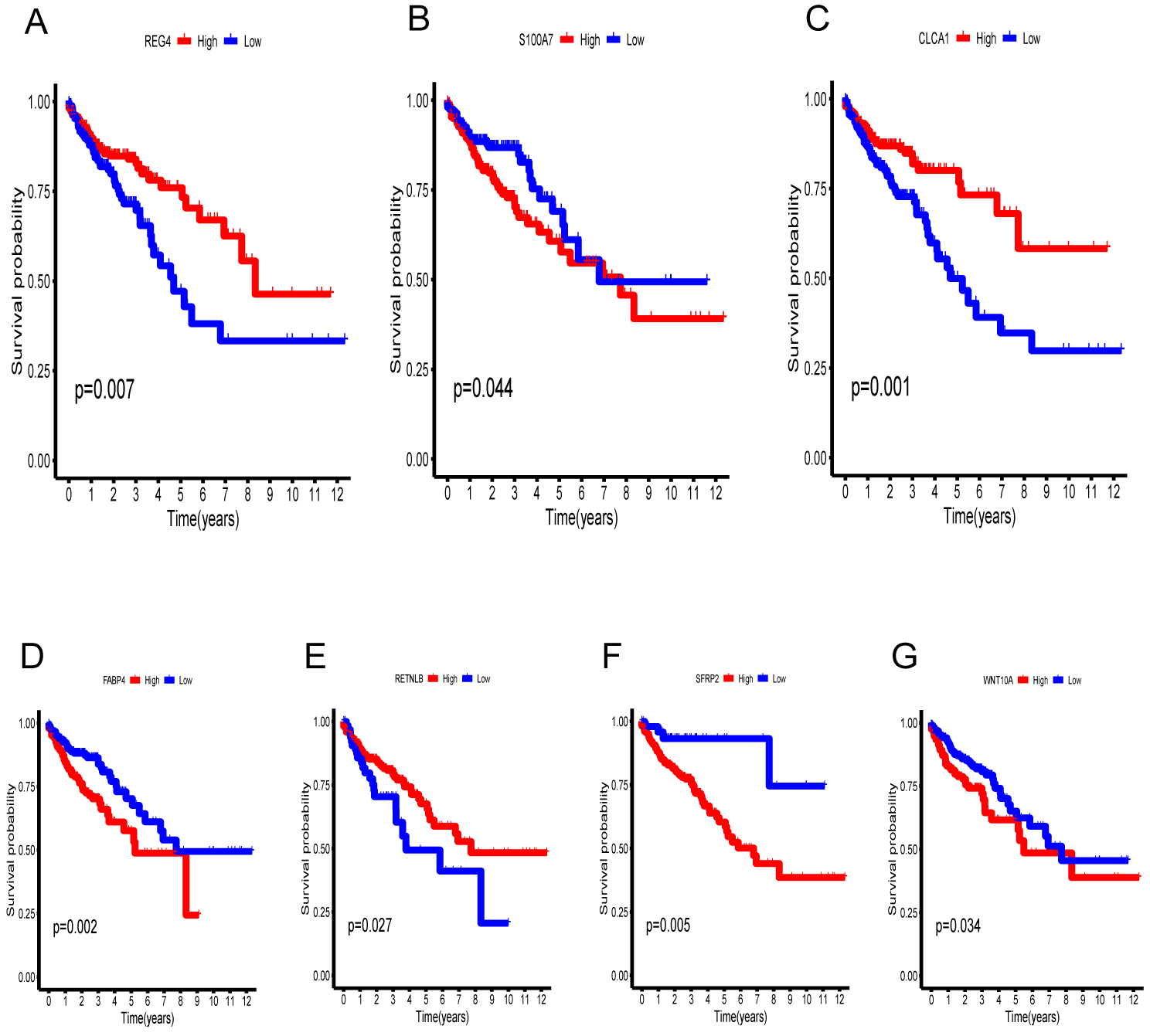

Supplement: Supplementary file 1 — Additional file 1: Figure S1. A ROC curves of the predictive efficiency in the test set. B and C Forest plots of univariate and multivariate Cox regression analyses in the test set. Fig. S2. PPI network of DEGs in the high and low risk score groups. Fig. S3. Expression of REG4， S100A7， CLCA1, FABP4, KRT14, RETNLB, SFRP2, and WNT10A in TCGA CC and normal colon samples. Fig. S4. A–G OS of REG4, S100A7, CLCA1, FABP4, RETNLB, SFRP2, and WNT10A in TCGA. [file 12859_2023_5204_MOESM1_ESM.docx]
