# Supplementary material for: Seven bacterial response-related genes are biomarkers for colon cancer
Source: BMC Bioinformatics. 2023 Mar 20;24:103. doi: 10.1186/s12859-023-05204-4 (PMC10026208; doi:10.1186/s12859-023-05204-4)
Supplement: Supplementary file 6 — Additional file 6. qPCR methods and the primers (Sangon) used for 9 genes qPCR. [file 12859_2023_5204_MOESM6_ESM.docx]

**Additional file 6: qPCR methods and the primers (Sangon) used for 9 genes qPCR**

qPCR methods

Twenty-seven patients with matched adjacent normal and CC tissue samples were enrolled in this study between April 2022 to September 2022 in the Department of Colorectal Surgery, First Affiliated Hospital of Guangxi Medical University (Guangxi Zhuang Autonomous Region, China). These patients were diagnosed CC and underwent surgery at the hospital. The CC cell line COLO320DM was purchased from Procell Life Science & Technology Co., Ltd. (Wuhan, Hubei, China). The CRC cell line SW480 was purchased from Servicebio (Wuhan, Hubei, China). The normal colorectal cell line FHC were purchased from Bluebio (Shanghai, China). All cell lines were verified using STR. All aspects of this study were approved by the Ethics Committee of the First Affiliated Hospital of Guangxi Medical University (Aproval Number:2022-E415-01). Informed consent was obtained from all the participants.

*LGALS4, RORC, DDIT3, NSUN5, RBCK1, RGL2,* and *SERPINE1* and their coexpression with lncNRAs C6orf223 and SLC12A9-AS1 were verified in colon tissues. *LGALS4* and *NSUN5* expressions were also verified in cell lines. High-quality total RNA was extracted from tissues and cells using TRIzol reagent (No. B511321; Sangon Biotech, Shanghai, China). M-MuLV First Strand cDNA Synthesis Kit (No. B532435, Sangon Biotech) was used to conduct complementary DNA synthesis. 2X SYBR Green Abstart PCR Mix (No. B110031; Sangon Biotech) was used to test gene expression using a Thermo Fisher Real-Time PCR System (Applied Biosystems, Thermo Fisher Scientific). The relative gene expression was calculated using the 2^-ΔΔCt^ method.

The primers (Sangon) used are as following:

*LGALS4*: Forward, CGACGCTGCCTTACTACCAG; Reverse, CCAACCACAAAGTTCACGAAGA.

*RORC*: Forward, AGTAGAACAGCTGCAGTACAAT; Reverse, CTGAAGAGCTCCTTGTAGAGTG.

*DDIT3*: Forward, GAACGGCTCAAGCAGGAAATC; Reverse, TTCACCATTCGGTCAATCAGAG.

*NSUN5*: Forward, CGCTACCATGAGGTCCACTAC; Reverse, GCATCTCGCACCACGTCTT.

*RBCK1*: Forward, TGTGTGTTTCCACGTCAACTG; Reverse, AGCCACATCGTTCTGAGCC.

*RGL2*: Forward, CTCATCCGCAATCTCCGGTC; Reverse, AAAGTTCCGCATCTAGCAGGG.

*SERPINE1*: Forward, ACCGCAACGTGGTTTTCTCA; Reverse, TTGAATCCCATAGCTGCTTGAAT.

C6orf223: Forward, GCCATGAGACTAGAGGTTGCTTGAG; Reverse, GAATCGGAGACACAGCCTTGCC.

SLC12A9-AS1: Forward, AGATGGCGGCGACTATGATG; Reverse, GGACGCAACCGCAGAAAAAG.

*GAPDH*: Forward, CAGGAGGCATTGCTGATGAT; Reverse, GAAGGCTGGGGCTCATTT.
