# Supplementary material for: Seven bacterial response-related genes are biomarkers for colon cancer
Source: BMC Bioinformatics. 2023 Mar 20;24:103. doi: 10.1186/s12859-023-05204-4 (PMC10026208; doi:10.1186/s12859-023-05204-4)
Supplement: Supplementary file 7 — Additional file 7. Data or codes for figure 4–7. [file 12859_2023_5204_MOESM7_ESM.zip › Additional file 7 Data or codes for figure 4-7/Data for Figure 5(A-E)/pRRophetic_0.5.tar.gz!/pRRophetic/inst/doc/vignetteOutline.pdf]

pRRophetic User's guide

Dr. Paul Geeleher

November 4, 2016

This file will demonstrate some use cases for the pRRophetic R packages. The package allows prediction of a phenotype from gene expression data. Here, we demonstrate the primary use case, which is prediction of clinical outcome using the Cancer Genome Project (CGP) cell line data. We also show that the package can be used for prediction of drug sensitivity in an external panel of cell lines (the Cancer Cell Line Encyclopedia (CCLE)) . Furthermore, we demonstrate that, at least in principle, the package can be used for prediction from clinical data (rather than just cell line data).

First, load the library.

```
> library(pRRophetic)
> library(ggplot2)
> set.seed(12345)
```

1 Predicting clinical outcome from the CGP cell lines

The primary use case of pRRophetic is predicting clinical outcome to chemotherapy, from baseline tumor gene expression data. This is achieved using the CGP cell lines as a training set and

Load the bortezomib expression data. This is stored in a matrix with Gene Symbols as row names. Note that in order for ”pRRopheticPredict” to work, the matrix MUST be annotated with official gene symbols.

```
> data("bortezomibData") #exprDataBortezomib, bortIndex, studyResponse and studyIndex
```

Assess the normality of the data. For many of the drug in CGP, the data deviate wildly from a normal distribtion and should not be fit as the response variable in a linear model (i.e. pRRopheticPredict is based on linear ridge regression).

```
> pRRopheticQQplot("Bortezomib")
```

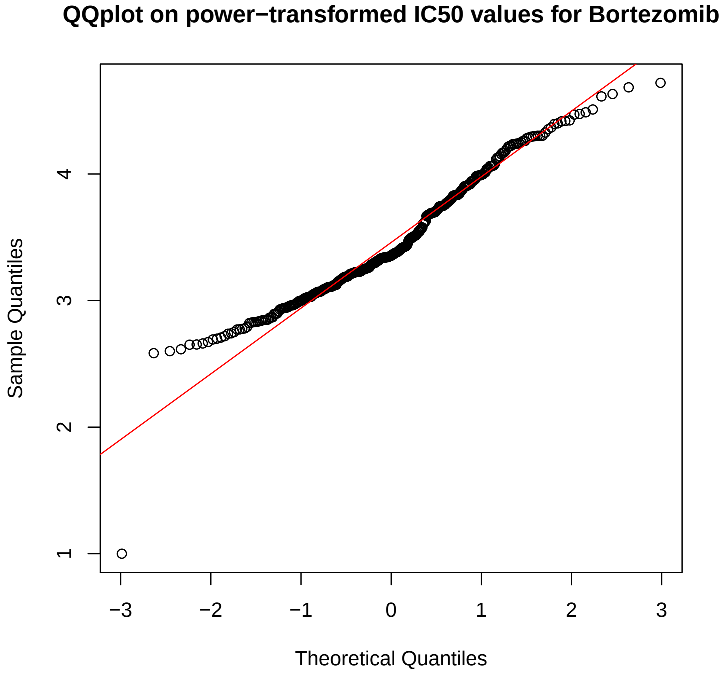

Perform 5-fold cross-validation on the training set (i.e. the CGP cell lines). This will give an indication of whether we may be able to predict clinical drug sensitivity.

```
> cvOut <- pRRopheticCV("Bortezomib", cvFold=5, testExprData=exprDataBortezomib)
```

```
 11683  gene identifiers overlap between the supplied expression matrices...

Found 2 batches
Adjusting for 0 covariate(s) or covariate level(s)
Standardizing Data across genes
Fitting L/S model and finding priors
Finding parametric adjustments
Adjusting the Data

1 of 5 iterations complete.
2 of 5 iterations complete.
3 of 5 iterations complete.
4 of 5 iterations complete.
5 of 5 iterations complete.
```

```
> summary(cvOut)
```

```
Summary of cross-validation results:

Pearsons correlation: 0.45 , P =  1.33226762955019e-15
R-squared value: 0.2
Estimated 95% confidence intervals: -4.16, 3.35
Mean prediction error: 1.62
```

Plot the cross validation predicted phenotype against the measured IC50s.

```
> plot(cvOut)
```

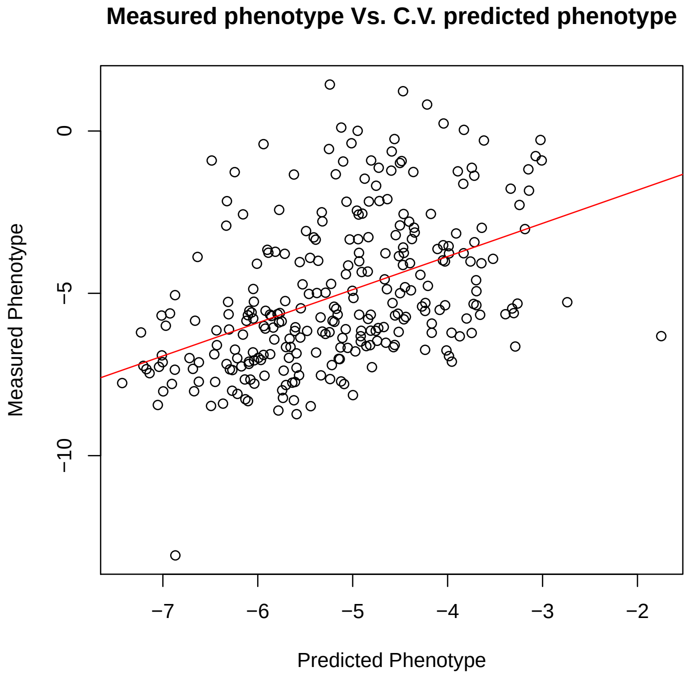

Based on the qqplot it is likely acceptable to use these data for prediction of bortezomib sensitivity. Predict bortezomib sensitivity using all cell lines, then only cell lines from hematological cancers and then only cell lines from derived from solid tumors.

```
> predictedPtype <- pRRopheticPredict(exprDataBortezomib, "Bortezomib",
+ selection=1)
```

```
 11683  gene identifiers overlap between the supplied expression matrices...

Found 2 batches
Adjusting for 0 covariate(s) or covariate level(s)
Standardizing Data across genes
Fitting L/S model and finding priors
Finding parametric adjustments
Adjusting the Data

 2324 low variabilty genes filtered.
Fitting Ridge Regression model... Done

Calculating predicted phenotype...Done
```

```
> predictedPtype_blood <- pRRopheticPredict(exprDataBortezomib, "Bortezomib",
+ "blood", selection=1)
```

```
 11683  gene identifiers overlap between the supplied expression matrices...

Found 2 batches
Adjusting for 0 covariate(s) or covariate level(s)
Standardizing Data across genes
Fitting L/S model and finding priors
Finding parametric adjustments
Adjusting the Data

 2324 low variabilty genes filtered.
Fitting Ridge Regression model... Done

Calculating predicted phenotype...Done
```

```
> predictedPtype_solid <- pRRopheticPredict(exprDataBortezomib, "Bortezomib",
+ "allSolidTumors", selection=1)
```

```
 11683  gene identifiers overlap between the supplied expression matrices...

Found 2 batches
Adjusting for 0 covariate(s) or covariate level(s)
Standardizing Data across genes
Fitting L/S model and finding priors
Finding parametric adjustments
Adjusting the Data

 2324 low variabilty genes filtered.
Fitting Ridge Regression model... Done

Calculating predicted phenotype...Done
```

Compare these three types of models. Interestingly, models trained on only ”blood” cancer cell lines perform best.

```
> t.test(predictedPtype[((studyResponse == "PGx_Responder = NR") & bortIndex)],
+ predictedPtype[((studyResponse == "PGx_Responder = R") & bortIndex)],
+ alternative="greater")
```

```
        Welch Two Sample t-test

data:  predictedPtype[((studyResponse == "PGx_Responder = NR") & bortIndex)] and predicted
t = 3.0109, df = 163.75, p-value = 0.001509
alternative hypothesis: true difference in means is greater than 0
95 percent confidence interval:
 0.1826465       Inf
sample estimates:
mean of x mean of y
-4.926576 -5.331926
```

```
> t.test(predictedPtype_blood[((studyResponse == "PGx_Responder = NR") & bortIndex)],
+ predictedPtype_blood[((studyResponse == "PGx_Responder = R") & bortIndex)],
+ alternative="greater")
```

```
        Welch Two Sample t-test

data:  predictedPtype_blood[((studyResponse == "PGx_Responder = NR") &  and predictedPtype
t = 4.1204, df = 165.24, p-value = 2.984e-05
alternative hypothesis: true difference in means is greater than 0
95 percent confidence interval:
 0.3975589       Inf
sample estimates:
mean of x mean of y
-4.372173 -5.036370
```

```
> t.test(predictedPtype_solid[((studyResponse == "PGx_Responder = NR") & bortIndex)],
+ predictedPtype_solid[((studyResponse == "PGx_Responder = R") & bortIndex)],
+ alternative="greater")
```

```
        Welch Two Sample t-test

data:  predictedPtype_solid[((studyResponse == "PGx_Responder = NR") &  and predictedPtype
t = 1.1167, df = 165.87, p-value = 0.1329
alternative hypothesis: true difference in means is greater than 0
95 percent confidence interval:
 -0.07325769         Inf
sample estimates:
mean of x mean of y
-5.381191 -5.533412
```

Estimate the PPN and PPV values for the predictions above. A cutpoint is estimated from the mean IC50 value in the training data.

```
> allTissuesPpv <- getPPV(predResponders=predictedPtype[((studyResponse == "PGx_Responder
```

```
PPV:  0.57
NPV:  0.59
Cutpoint:  -5.04
```

```
> bloodPpv <- getPPV(predResponders=predictedPtype_blood[((studyResponse == "PGx_Responder
```

```
PPV:  0.63
NPV:  0.69
Cutpoint:  -4.54
```

Make a boxplot of the results of the blood-only model.

```
> df <- stack(list(NR=predictedPtype_blood[((studyResponse == "PGx_Responder = NR")
+ & bortIndex)], R=predictedPtype_blood[((studyResponse == "PGx_Responder = R") &
+ bortIndex)]))
> ggplot(data=df, aes(y=values, x=ind)) + geom_boxplot(alpha=.3, fill=c("#CC0033", "#00663
+ theme_bw() + ylab("Predicted Bortezomib Sensitivity") + xlab("Clinical Response")
```

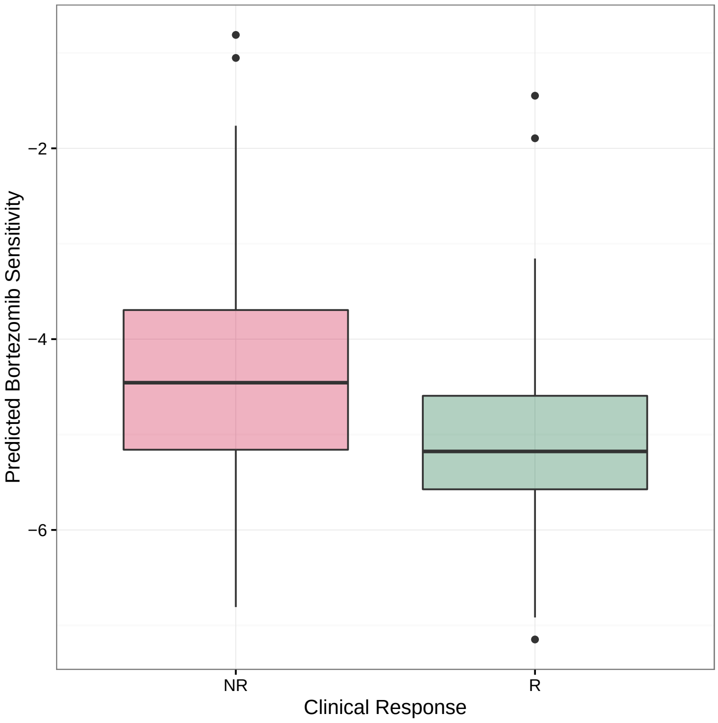

2 Predict drug sensitivity in CCLE. Demonstrate both linear and logistic models

Lets predict for PD0332991 using pRRopheticPredict(). First, load the CCLE expression and phenotype data. This loads two objects sensDataCcle and exprMatCcle.

```
> data(ccleData) #sensDataCcle, exprMatCcle
```

Do 10 fold cross-validation on this drug.

```
> cvOut_pd <- pRRopheticCV("PD.0325901", cvFold=5, testExprData=exprMatCcle)

 11897  gene identifiers overlap between the supplied expression matrices...

Found 2 batches
Adjusting for 0 covariate(s) or covariate level(s)
Standardizing Data across genes
Fitting L/S model and finding priors
Finding parametric adjustments
Adjusting the Data

1 of 5 iterations complete.
2 of 5 iterations complete.
3 of 5 iterations complete.
4 of 5 iterations complete.
5 of 5 iterations complete.
```

```
> summary(cvOut_pd)

Summary of cross-validation results:

Pearsons correlation: 0.57 , P =  0
R-squared value: 0.33
Estimated 95% confidence intervals: -3.68, 3.63
Mean prediction error: 1.44
```

Plot the cross-validation predicted phenotype against the measured phenotype.

```
> plot(cvOut_pd)
```

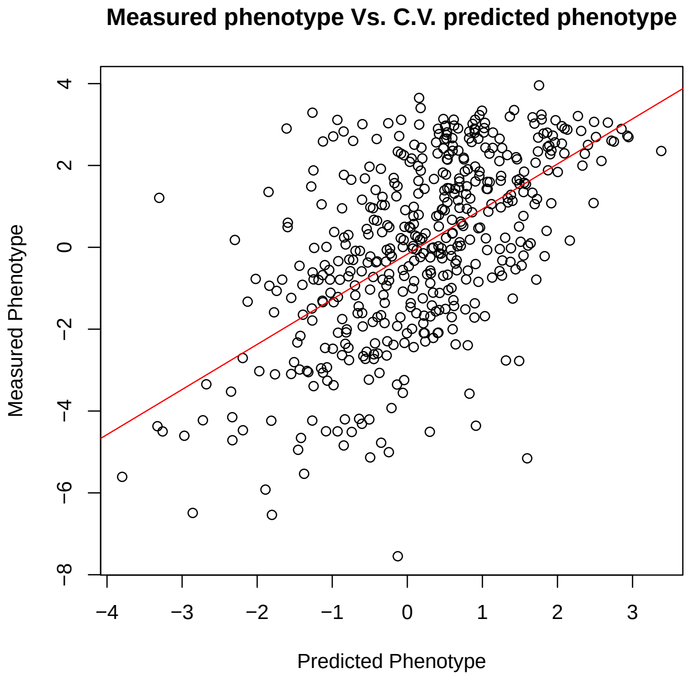

Run the prediction for PD0325901 on the CCLE data.

```
> predictedPtype_ccle <- pRRopheticPredict(exprMatCcle, "PD.0325901", selection=1)

 11897  gene identifiers overlap between the supplied expression matrices...

Found 2 batches
Adjusting for 0 covariate(s) or covariate level(s)
Standardizing Data across genes
Fitting L/S model and finding priors
Finding parametric adjustments
Adjusting the Data

 2380 low variabilty genes filtered.
```

```
Fitting Ridge Regression model... Done

Calculating predicted phenotype...Done
```

Get the ActArea (a measure of drug sensitivity) for the CCLE cell lines for which we have just predicted IC50. We will not use IC50 values as they have been capped at the maximum drug screening concentration in CCLE.

```
> cellLinesWithCcleIc50s <- names(predictedPtype_ccle)[names(predictedPtype_ccle) %in%
+ sensDataCcle$CCLE.Cell.Line.Name]
> predCcleOrd <- predictedPtype_ccle[names(predictedPtype_ccle)]
> ccleActArea_pd <- -sensDataCcle$"ActArea"[sensDataCcle$Compound == "PD-0325901"]
> names(ccleActArea_pd) <- sensDataCcle$"CCLE.Cell.Line.Name"[sensDataCcle$Compound ==
+ "PD-0325901"]
> ccleActAreaord <- ccleActArea_pd[cellLinesWithCcleIc50s]
```

Compare prediction to measured IC50, it is actually higher than the correlation achieved for remeasuring the drug sensitivity.

```
> cor.test(predictedPtype_ccle[cellLinesWithCcleIc50s], ccleActAreaord,
+ method="spearman")

        Spearman's rank correlation rho

data:  predictedPtype_ccle[cellLinesWithCcleIc50s] and ccleActAreaord
S = 7728300, p-value < 2.2e-16
alternative hypothesis: true rho is not equal to 0
sample estimates:
      rho
0.5807112
```

Plot the resulting correlation between predicted and measured values.

```
> df2 <- data.frame(predCcle=predictedPtype_ccle[cellLinesWithCcleIc50s],
+ actAreaCcle=ccleActAreaord)
> ggplot(data=df2, aes(y=predCcle, x=actAreaCcle)) + geom_point(alpha=0.5) +
+ geom_smooth(method=lm) + theme_bw() + xlab("Measured Activity Area") +
+ ylab("Predicted Drug Sensitivity")
```

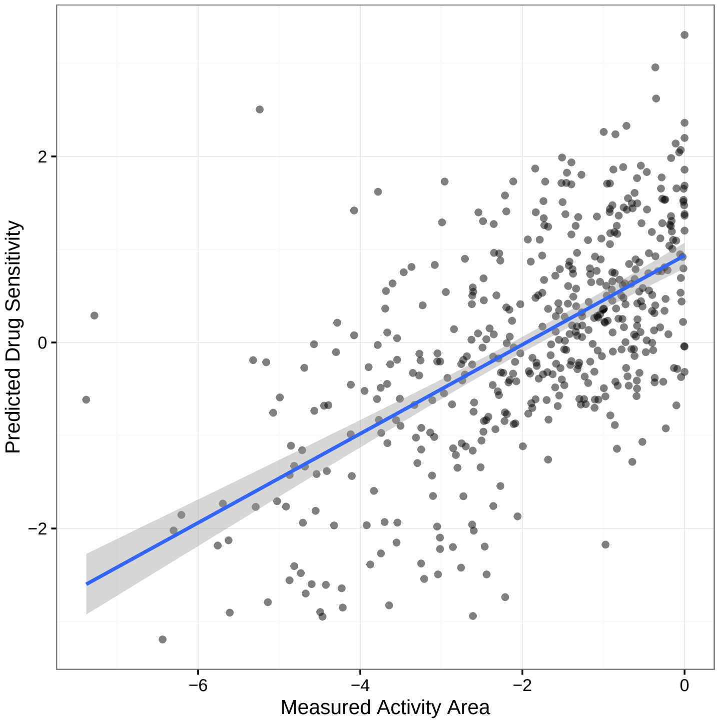

Next, do some prediction for Erlotinib, a targeted agent. To demonstrate how to create a logistic model, lets predict for erlotinib using pRRopheticLogisiticPredict(). This function will return the log odds of sensitivity

```
> predictedPtype_ccle_erlotinib <- pRRopheticLogisticPredict(exprMatCcle, "Erlotinib",
+ selection=1)
```

```
 11897  gene identifiers overlap between the supplied expression matrices...

Found 2 batches
Adjusting for 0 covariate(s) or covariate level(s)
Standardizing Data across genes
Fitting L/S model and finding priors
Finding parametric adjustments
Adjusting the Data
Fitting model, may take some time....
```

Get the ActArea for the CCLE cell lines for which we have just predicted IC50.

```
> cellLinesWithCcleIc50s <-
+ names(predictedPtype_ccle_erlotinib)[names(predictedPtype_ccle_erlotinib) %in%
+ sensDataCcle$CCLE.Cell.Line.Name]
> predCcleOrd <- predictedPtype_ccle_erlotinib[names(predictedPtype_ccle_erlotinib)]
> ccleActArea_pd <- sensDataCcle$"ActArea"[sensDataCcle$Compound == "Erlotinib"]
> names(ccleActArea_pd) <- sensDataCcle$"CCLE.Cell.Line.Name"[sensDataCcle$Compound ==
+ "Erlotinib"]
> ccleActAreaord <- ccleActArea_pd[cellLinesWithCcleIc50s]
```

There are a very large number of cell lines resistant to Erlotinib (within the drug screening window), so a correlation is not an appropriate measure of concordance. So lets do a t-test between some of the most sensitive and resistant cell lines to assess whether signal is being captured by the predictions.

```
> resistant <- names(sort(ccleActAreaord))[1:55] #55 highly resistant cell lines.
> sensitive <- names(sort(ccleActAreaord, decreasing=TRUE))[1:15] #15 sensitive
> t.test(predictedPtype_ccle_erlotinib[resistant],
+ predictedPtype_ccle_erlotinib[sensitive])

        Welch Two Sample t-test

data:  predictedPtype_ccle_erlotinib[resistant] and predictedPtype_ccle_erlotinib[sensitiv
t = -5.7674, df = 23.477, p-value = 6.574e-06
alternative hypothesis: true difference in means is not equal to 0
95 percent confidence interval:
 -7.125809 -3.366622
sample estimates:
mean of x mean of y
-6.600899 -1.354683
```

Despite the fact that IC50 values are not correlated for this drug between these studies, the most sensitive/resistant samples are separated highly significantly with this logistic models.

```
> boxplot(list(Resistant=predictedPtype_ccle_erlotinib[resistant],
+ Sensitive=predictedPtype_ccle_erlotinib[sensitive]), pch=20,
+ vertical=TRUE, method="jitter", ylab="Log-odds of sensitivity")
```

3 Clinical drug-sensitivity prediction from clinical data

Include, is an example, prediction from the bortezomib clinical data where we try to predict CR, PR, MR, NC, PD from CR, PR, MR, NC, PD. This serves as both an example of prediction directly from clinical data and of using a dataset other than the CGP from which to predict.

First, prepare the training data and test expression data.

```
> trainExpr <- exprDataBortezomib[, (detailedResponse %in% c(1,2,3,4,5)) &
+ studyIndex %in% c("studyCode = 25", "studyCode = 40")]
> trainPtype <- detailedResponse[(detailedResponse %in% c(1,2,3,4,5)) &
+ studyIndex %in% c("studyCode = 25", "studyCode = 40")]
> testExpr <- exprDataBortezomib[, (detailedResponse %in% c(1,2,3,4,5)) &
+ studyIndex %in% c("studyCode = 39")]
```

Calculate the predicted phenotype.

```
> ptypeOut <- calcPhenotype(trainExpr, trainPtype, testExpr, selection=1)

 22283  gene identifiers overlap between the supplied expression matrices...
```

```
Found 2 batches
Adjusting for 0 covariate(s) or covariate level(s)
Standardizing Data across genes
Fitting L/S model and finding priors
Finding parametric adjustments
Adjusting the Data

 2500 low variabilty genes filtered.
Fitting Ridge Regression model... Done

Calculating predicted phenotype...Done
```

We do capture some signal.

```
> testPtype <- detailedResponse[(detailedResponse %in% c(1,2,3,4,5)) &
+ studyIndex %in% c("studyCode = 39")]
> cor.test(testPtype, ptypeOut, alternative="greater")

        Pearson's product-moment correlation

data:  testPtype and ptypeOut
t = 2.1512, df = 139, p-value = 0.01659
alternative hypothesis: true correlation is greater than 0
95 percent confidence interval:
 0.04142448 1.00000000
sample estimates:
      cor
0.1795014
```

This t-test allows us to compare results directly to the cell line model, however, the cell line model outperforms this particular clinical model.

```
> t.test(ptypeOut[testPtype %in% c(3,4,5)], ptypeOut[testPtype %in% c(1,2)],
+ alternative="greater")

        Welch Two Sample t-test

data:  ptypeOut[testPtype %in% c(3, 4, 5)] and ptypeOut[testPtype %in% c(1, 2)]
t = 2.0182, df = 137.43, p-value = 0.02276
alternative hypothesis: true difference in means is greater than 0
95 percent confidence interval:
 0.02533599        Inf
sample estimates:
mean of x mean of y
 2.646449  2.505269

> sessionInfo()

R version 3.2.3 (2015-12-10)
Platform: x86_64-pc-linux-gnu (64-bit)
Running under: Ubuntu 14.04.3 LTS
```

```
locale:
 [1] LC_CTYPE=en_US.UTF-8       LC_NUMERIC=C
 [3] LC_TIME=en_US.UTF-8        LC_COLLATE=C
 [5] LC_MONETARY=en_US.UTF-8    LC_MESSAGES=en_US.UTF-8
 [7] LC_PAPER=en_US.UTF-8       LC_NAME=C
 [9] LC_ADDRESS=C               LC_TELEPHONE=C
[11] LC_MEASUREMENT=en_US.UTF-8 LC_IDENTIFICATION=C

attached base packages:
[1] stats     graphics  grDevices utils     datasets  methods   base

other attached packages:
[1] ggplot2_2.1.0  pRRophetic_0.5

loaded via a namespace (and not attached):
 [1] Rcpp_0.12.1          plyr_1.8.3           nloptr_1.0.4
 [4] GenomeInfoDb_1.4.3   tools_3.2.3          lme4_1.1-10
 [7] annotate_1.46.1      RSQLite_1.0.0        preprocessCore_1.30.0
[10] nlme_3.1-122         gtable_0.1.2         lattice_0.20-33
[13] mgcv_1.8-7           Matrix_1.2-2         DBI_0.3.1
[16] parallel_3.2.3       SparseM_1.7          genefilter_1.50.0
[19] S4Vectors_0.6.6      MatrixModels_0.4-1   IRanges_2.2.9
[22] stats4_3.2.3         grid_3.2.3           nnet_7.3-11
[25] Biobase_2.28.0       AnnotationDbi_1.30.1 XML_3.98-1.3
[28] survival_2.38-3      sva_3.14.0           minqa_1.2.4
[31] car_2.1-0            scales_0.3.0         MASS_7.3-44
[34] splines_3.2.3        BiocGenerics_0.14.0  pbkrtest_0.4-2
[37] ridge_2.1-3          xtable_1.7-4         colorspace_1.2-6
[40] labeling_0.3         quantreg_5.19        munsell_0.4.2
```
