# Supplementary material for: Seven bacterial response-related genes are biomarkers for colon cancer
Source: BMC Bioinformatics. 2023 Mar 20;24:103. doi: 10.1186/s12859-023-05204-4 (PMC10026208; doi:10.1186/s12859-023-05204-4)
Supplement: Supplementary file 7 — Additional file 7. Data or codes for figure 4–7. [file 12859_2023_5204_MOESM7_ESM.zip › Additional file 7 Data or codes for figure 4-7/Data for Figure 5(A-E)/pRRophetic_0.5.tar.gz!/pRRophetic/vignettes/vignetteOutline-008.pdf]

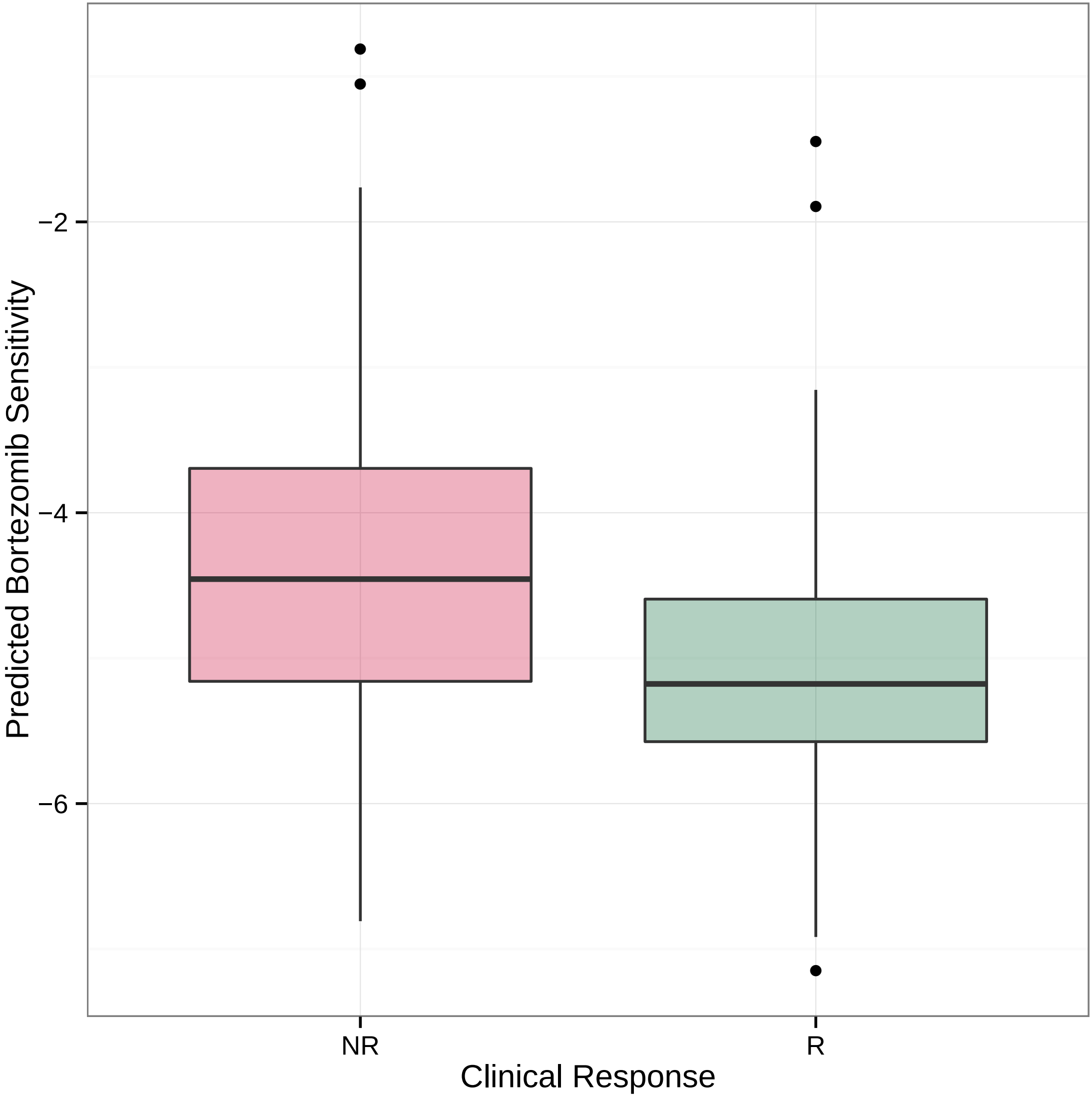

−2
−4
−6
Predicted Bortezomib Sensitivity
NR
R
Clinical Response
