# Supplementary material for: Seven bacterial response-related genes are biomarkers for colon cancer
Source: BMC Bioinformatics. 2023 Mar 20;24:103. doi: 10.1186/s12859-023-05204-4 (PMC10026208; doi:10.1186/s12859-023-05204-4)
Supplement: Supplementary file 7 — Additional file 7. Data or codes for figure 4–7. [file 12859_2023_5204_MOESM7_ESM.zip › Additional file 7 Data or codes for figure 4-7/Data for Figure 7B/immFunction.pdf]

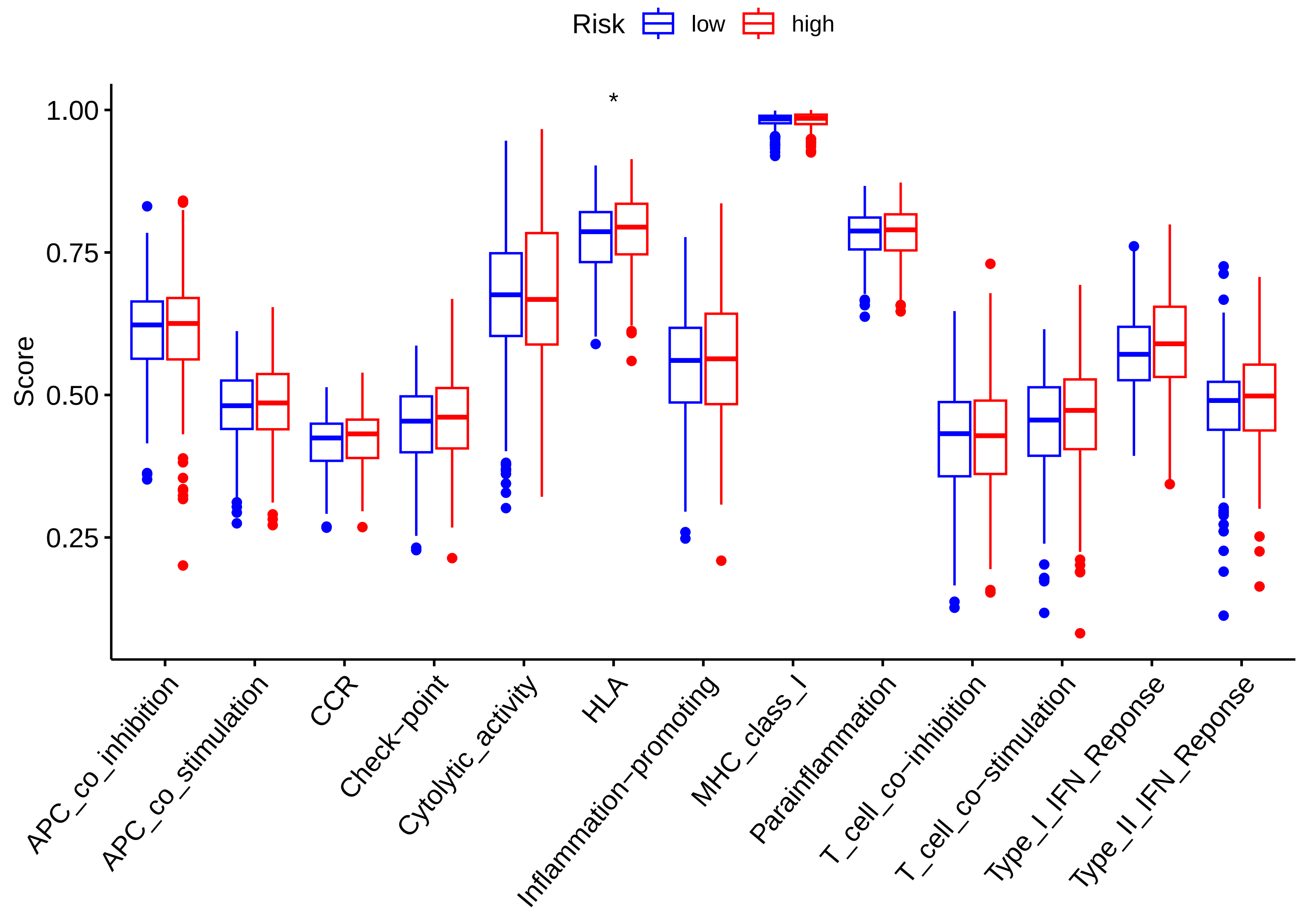

Risk
low
high
Score
1.00
0.75
0.50
0.25
*
APC_co_inhibition
APC_co_stimulation
CCR
Check−point
Cytolytic_activity
HLA
Inflammation−promoting
MHC_class_I
Parainflammation
T_cell_co−inhibition
T_cell_co−stimulation
Type_I_IFN_Reponse
Type_II_IFN_Reponse
